# Double Negative (DN) B cells: A connecting bridge between rheumatic diseases and COVID-19?

**DOI:** 10.31138/mjr.32.3.192

**Published:** 2021-09-30

**Authors:** Athanasios Sachinidis, Alexandros Garyfallos

**Affiliations:** 4^th^ Department of Internal Medicine, Hippokration General Hospital, School of Medicine, Aristotle University of Thessaloniki, Thessaloniki, Greece

**Keywords:** Double negative B cells, DN2, rheumatic diseases, lupus, COVID-19, pandemic

## Abstract

Double Negative (DN) B cells constitute a B cell population that lacks expression of immunoglobulin D and CD27 memory marker. These cells expand in elderly healthy individuals, but also accumulate prematurely in autoimmune and infectious diseases.

COVID-19 is a pandemic infectious disease caused by SARS-CoV-2, a coronavirus that was first observed in Wuhan, China in December 2019. In its more severe cases, COVID-19 causes severe pneumonia and acute respiratory syndrome with high morbidity and mortality.

Recent studies have revealed that the extrafollicular DN2 B cell subset, previously described in lupus patients, does also expand in severe and/or critical groups of COVID-19 patients. These DN2 cells correlate with disease severity and laboratory parameters of inflammation. However, their exact role and function in COVID-19 require to be further investigated.

In this review, we highlight the DN immune responses in both rheumatic diseases and COVID-19, and we point out the importance of clarifying DN’s role in the immunopathology of the aforementioned infection, as it could probably enable better management of rheumatic diseases during the pandemic. Of note, the symptomatology of COVID-19, as well as the potential outcome of death, have given rise to a worldwide concern and scare of exposition to SARS-CoV-2, especially among the rheumatological patients who believe to be at higher risk due to their immunological background and the immunosuppressive therapies. Nevertheless, there is no convincing evidence so far that these patients are truly at higher risk than others.

## INTRODUCTION

Coronavirus disease 2019 (COVID-19) is an infectious disease caused by severe acute respiratory syndrome coronavirus 2 (SARS-CoV-2), a single stranded RNA virus that was first observed in Wuhan, China in December 2019. Since then, the disease has emerged as a pandemic.^1^ In most individuals, the infection is considered as asymptomatic or paucisymptomatic, while in some cases it causes severe pneumonia, myocarditis, acute kidney injuries, hepatitis, acute respiratory distress syndrome (ARDS), multiorgan failure and even death.^1^

The most severe cases of COVID-19 are characterised by a cytokine storm which induces hyperinflammation, and lymphopenia, which affects both the innate and adaptive immune responses.^2^ Current evidence suggests that the hyperinflammatory syndrome in COVID-19 patients results from a dysregulated host innate immune response.^3,4^ Furthermore, the composition of the surviving T lymphocytes, as significant reductions in peripheral T cells have been reported in the severe cases of COVID-19,^5^ seems to be important for the establishment of the hyperinflammatory state.^3,4^ Having taken into account these facts, most studies so far have focused on the innate immune system responses and the phenotypic and/or molecular T-cell alterations.

On the other hand, the knowledge regarding the immunology of B cells in COVID-19 is limited. However, a few studies have turned their attention to humoral immune responses, and thus investigated the role and function of B cells in the infection.^6–9^ As in many other infectious diseases such as HIV, hepatitis B and malaria,^9,10,11^ alterations of the B cell compartment have also been reported in COVID-19. ^6–9,12^ Interestingly, analyses of B cell populations revealed that extrafollicular double negative (DN) B cells, a characteristic of active systemic lupus erythematosus (SLE),^13^ are a prominent feature of severe COVID-19 as well.^7,12,14,15^ Moreover, the frequencies of these DN cells correlate with disease severity profiles and some clinical and laboratory parameters of the disease.^6–8,14^ Their exact role and function though in COVID-19 is not yet fully understood.

In this review, we discuss the overlapping DN immune responses in rheumatic diseases and severe COVID-19, and we point out the importance of investigating the role and the function of DN B cells in the immunopathology of the aforementioned infection, as it could probably enable better management of rheumatological patients during the pandemic.

## THE POPULATION OF DOUBLE-NEGATIVE (DN) B CELLS

Double negative (DN) B cells, also known as atypical memory B cells, constitute a B cell population that lacks expression of immunoglobulin D and CD27 memory marker.^16^ The population is expanded in the elderly,^16^ but also represents a notable component of the B cell compartment in patients with autoimmune and/or chronic infectious diseases.^17–20^ In general, DN B cells seem to serve different functions in the context of different conditions. For instance, in cases of HIV and malaria, these cells have been characterised as having an exhausted phenotype,^19,20^ while in cases of SLE they are poised to become antibody-secreting cells (ASCs) in response to TLR7 ligands and the cytokines IFN-γ and IL-21,^13^ and are considered to be the direct source of ASCs and serum autoantibodies during disease’s flares.^13, 21–23^ Such a differing and condition-dependent functionality of DN B cells, probably can be justified by the heterogeneity of the DN population.

### Heterogeneity among DN B cells: subsets and DN relationship to other B cells arising in autoimmunity and infections

Various names have been used to describe CD19^+^IgDCD27^−^ cells, such as age-associated B cells (ABCs), atypical memory B cells, CD11c^hi^ B cells and of course double negative B cells.^16,18,20,21^ Truth is that all these terms referred to B cell populations that share a number of features and are enriched in the peripheral blood of both elderly healthy individuals and people with chronic infectious diseases or inflammatory conditions, such as autoimmune disorders, obesity and cancer.^16–20,24–26^ A recent study however, based on transcriptomic and IgH repertoire analyses of B cells, questions the equivalence of all these populations.^27^ Here, we shall discuss the DN B cell population and its subsets, as well as the DN relationship to other B cells arising in autoimmunity and infections.

#### The various DN B cell subsets

In 2018, Jenks and colleagues from the Sanz group were the first to divide DN B cells into two subgroups, based on the expression of follicular homing marker CXCR5.^13^ Up to this time, the term DN B cells had been used to describe all B cell populations that lack expression of IgD and CD27. However, according to Jenks and his colleagues, the CXCR5^+^ subgroup, now known as DN1, is the DN sub-population that expands in elderly healthy individuals,^16^ while the CXCR5- subgroup constitutes the extrafollicular DN2 subset that is more marked in active SLE.^13,17^ Furthermore, transcription factor T-bet, whose expression in B cells is considered to be of high importance for the induction of autoimmunity and/or the control of chronic infections,^28,29^ is expressed (now obviously) in DN2 subset only.^13^

Of note, the frequencies of DN2 B cells in rheumatoid arthritis (RA), primary Sjögren’s syndrome (pSS) and scleroderma (SCD) patients were not as much elevated as in SLE patients.^13^ Thus, the subset was presumed to be SLE specific. However, subsequently published data has implicated DN2 in the pathogenesis of common variable immunodeficiency (CVID) as well.^30^ Moreover, despite the fact that no classification into DN1 or DN2 has been achieved in incidents involving obese people, the DN B cells in obese individuals are believed to be predominantly DN2, as they secrete anti-self IgG antibodies.^24,25^

In addition, a cytoplasmic FOXO1 DN population - termed cytoFOX DN B cells - was identified in SLE via deep immunophenotyping.^31^ FOXO1, which is a transcription factor involved in B cell development,^32^ translocates from the nucleus to the cytoplasm in response to BCR ligation and presumably in an AKT-dependent manner, and gets inactivated.^33^ The cytoplasmic localization of the transcription factor seems to serve as a biomarker of SLE progression.^31^ Considering the fact that highest frequencies of cytoFOX DN B cells have been observed in African-American female SLE patients, made us believe -up to this day- that these cells belong to the DN2 subset,^34^ as DN2 cells predominate in African-American SLE patients (especially females).^13^ However, we still cannot rule out the possibility that cytoFOX DN cells are equivalent to another “new” DN subset in SLE.

Taking into account all these data, we believe that DN B cells that have been reported in inflammatory conditions other than SLE and obesity, such as multiple sclerosis (MS),^18^ display a strong possibility to belong to the DN2 subset. However, we note that no confirmatory evidence exists thus far regarding this issue, as functional, transcriptional, phenotypic and other types of analyses need to be done. For instance, our knowledge regarding the presence of DN B cells in early-stage non-small cell lung cancer (NSCLC) is extremely limited at this time.^26^

As far as infectious diseases are concerned, DN B cells seem to play an inhibitory role in the humoral responses.^19,20,35,36^ Actually, these cells express a variety of inhibitor receptors, such as FcRL4, and exhibit many features of exhaustion,^19,20,35,36^ in contrast to DN2 B cells, which are very active in SLE.^13,21–23^ Moreover, the absence of FcRL4 in SLE DN B cells,^13,17^ further suggests that DN B cells in infectious diseases differ from DN2 cells. However, once again, no confirmatory evidence exists regarding this issue. Thus, as long as we cannot classify DN B cells in chronic infections into either DN1 or DN2 cells, we prefer to refer to them as atypical or tissue-based memory B cells.

We have already mentioned that, apart from SLE cases, DN2 cells have also been observed in severe and/or critical cases of COVID-19,^7,12,14,15^ and the frequencies of these cells correlate with the severity of the disease, as well as with some clinical and laboratory parameters, such as CRP, IL-6, and ferritin levels.^6–8,14^ Intriguingly, T-bet expression in DN2 cells from COVID-19 patients, was reduced as compared with healthy individuals,^6^ suggesting that these cells are probably not a major driver of inflammation in this disease but is more likely to play an inhibitory role in the regulation of T cell responses. We have to note though that the two cohorts analysed for T-bet expression, consisted of three COVID-19 patients and four healthy controls, respectively.^6^ We believe that a new study including more individuals from both two cohorts needs to be carried out, in order for these findings to be further strengthened.

Even more interestingly, a previously unreported DN B cell subset was detected in COVID-19 patients. This novel subset, now known as DN3, is defined by the absence of CD21 and CD11c, and seems to be associated with extrafollicular immune responses, along with DN2 cells.^7^ Of note, in comparison to DN2, DN3 cells display a broader significant correlation pattern with laboratory features that have been related to critical COVID-19.^8^ In general though, the subset is considered as entirely new and not yet characterised.

The main features and functions of DN B cells in rheumatic diseases and COVID-19 shall be described, in more detail, in next sections of this review. Nevertheless, a table summarizing the topics discussed above is provided here (**Table 1**).

**Table 1.** Discrete double-negative (DN) B cell subsets.

**Nomenclature**	**Phenotype**	**Prominence in (condition)**	**Properties**	**References**
**DN1**	CD19^+^IgD^−^CD27^−^CD21^+^ CD11c^−^ T-bet^−^ CXCR5^+^	Elderly healthy individuals (aging)	Memory precursors	[16,17]
**DN2**	CD19^+^IgD^−^CD27^−^CD21^−^ CD11c^+^ T-bet^+^CXCR5^−^	SLE (autoimmunity)	Extrafollicular ASC precursors	[17]
**DN3**	CD19^+^IgD^−^CD27^−^CD21^−^ CD11c^−^ [T-bet^low^]	COVID-19	??	[7,8]
**atMEM/tbMEM**	CD19^+^IgD^−^CD27^−^ CD21^−^FcRL4^+^	Chronic infections	Exhausted, mucosal resident B cells	[35,36]

DN1, DN2 and DN3: first, second and third subset of DN B cells, respectively; atMEM/tbMEM: atypical/tissue-based memory B cells; SLE: Systemic Lupus Erythematosus; ASC: antibody-secreting cell.

#### DN relationship to age-associated B cells (ABCs)

ABCs are a CD11c^+^T-bet^+^ B cell subset that expands continuously with age in healthy individuals.^37^ In cases of autoimmune and/or infectious diseases though, these cells accumulate prematurely and contribute to auto-antibody or anti-pathogen antibody production, respectively.^38–41^ Due to overlapping phenotypic markers, as well as similar activation requirements and functionality, ABCs and DN2 B cells are considered by many -including us- as relative populations.^13, 21,34,42,43^ However, a recent study by Maul et al., in which a comparative transcriptomic analysis was performed, suggests that ABCs are distinct from other CD11c^+^ B cell populations, such as DN2 cells, as they display an elevated expression in multiple cytokines and chemokines, not detected as increased in the other CD11c^+^ subsets. ^27^

Although we do not call into question the results of this study, we believe that the discrimination of ABCs from other CD11c^+^ populations came up from the use of a strict immunophenotype for the ABCs. More specifically, Maul et al. used the term “ABCs” to refer to murine spleen cells, defined as B220^+^CD19^+^CD11b^+^, that may express CD11c,^27^ while in the literature, consensus has not yet been reached as to which phenotypic markers should be used to properly define these cells.^34^ The heterogeneity of the markers used in different publications for the immunophenotyping of ABCs had previously been summarised in a review article by Phalke and Marrack.^42^ In that review, it becomes apparent that some scientific groups use the term “ABCs” to refer to human circulating B cells or even mouse circulating B cells that differ from the B220^+^CD19^+^CD11b^+^ murine spleen cells, but display a similar immunophenotype to DN2 cells (as defined in **Table 1** of this review article).^42^ Taking into consideration all the above, we still believe that ABCs and DN2 cells are related to each other, especially when we treat the first as non-murine cells only, but also as human cells,^18,21,38^ that express CD11c and T-bet, and lack expression of CD21. ^34^

## EXTRAFOLLICULAR B CELL RESPONSES IN RHEUMATIC DISEASES AND COVID-19

Germinal centres (GCs) are sites within the secondary lymphoid organs, in which somatically mutated high-affinity memory B cells and long-lived plasma cells are generated,^44^ thus sustaining immune protection and rapid recall responses against re-encountered antigens. In this process, apart from the proliferating antigen-specific B cells, the participation of follicular helper T cells and specialised follicular dendritic cells is also required.^44^ Although the GC reactions have remained the focus of B cell research, extrafollicular B cell responses - development of antibodies outside of the B cell follicles, in the absence of GCs and independently of follicular T cells - are nowadays of great interest to many scientists.^45,46^

**Figure 1.**
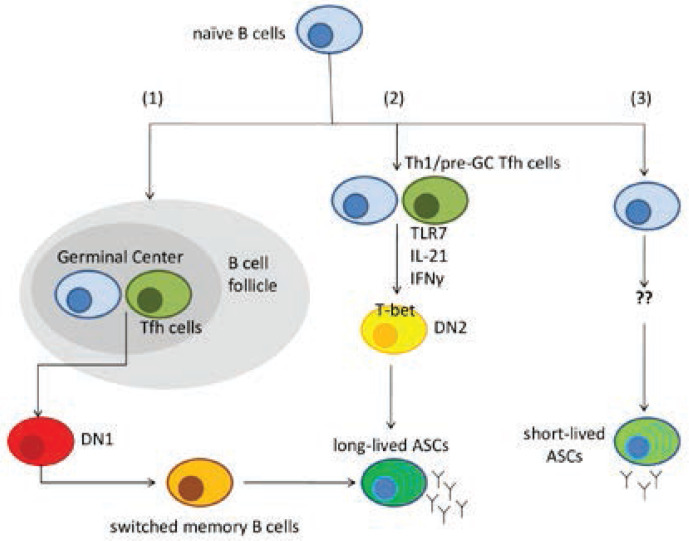
Germinal centre (GC) and extrafollicular B cell activation pathways. (**1**): In GC reactions, naïve B cells interact with T follicular helper cells (Tfh), thus generating class-switched memory B cells and long-lived antibody-secreting cells (ASCs). DN1 cells are considered as precursors of the memory cells. (**2**): Outside of the B cell follicles, naïve B cells may interact with peripheral helper T cells or pre-GC Tfh cells and differentiate, via the synergistic triggering of TLR7, IL-21 and IFNγ receptors, into DN2 cells that highly express transcription factor T-bet. DN2 cells serve as progenitors of ASCs with long lifespan. (**3**) An alternative T cell – independent extrafollicular pathway, involves naïve B cells that give rise to short-lived ASCs.

Traditionally, the extrafollicular B cell responses, in contrast to GC reactions, are thought to produce antibodies with low somatic mutations and develop short-lived immunological memory, in a T cell - independent manner.^47,48^ Subsequent findings though, revealed that peripheral helper T cells (or pre-GC follicular T cells) may contribute, under persistent antigen stimulation combined with Type I IFN signalling and innate-receptor sensing,^13^ to the induction of extrafollicular responses in patients with rheumatic diseases and/or infections.^7,23,49,50^ In these cases, B cells produced are class-switch recombination competent and capable of sustaining long-lived immunological responses.^7,23,48^ However, a key question which remains unanswered yet is whether these B cells which lead to exaggerated extrafollicular responses, such as DN2 cells reported in SLE and COVID-19,^7,13^ are drivers of disease pathogenesis or just a consequence of the strong inflammation that occurs. The GC and extrafollicular pathways (both T-independent and T-dependent) are presented in **Figure 1**.

### DN2 B cells in rheumatic diseases: emerging lessons from SLE

DN2 B cells are a unique double-negative population (IgD^−^CD27^−^) that displays extrafollicular characteristics, such as the lack of CXCR5 and CD62L expression.^13^ These cells are developmentally related to activated naïve B cells. However, they are considered as a distinct B cell population due to their unique transcriptomic profile and the reported increased rates of class-switch recombination.^13^ DN2 cells are hyper-responsive to TLR7 and, according to transcriptomic analyses, they seem to express a variety of cytokines and cytokine receptors, transcription factors such as T-bet, signalling factors and others.^13^

In severe cases of SLE, particularly in African-American female patients characterised by lupus nephritis and high titres of autoantibodies, DN2 cells expand dramatically and lead to the generation of autoreactive ASCs. In fact, the DN2 subset often becomes the predominant B cell population in these patients, with the highest frequencies of them being observed in the youngest individuals.^13^

In order for DN2 to be activated and differentiate into ASCs, TLR7 cooperates with IL-21 and IFNγ receptors. The synergistic triggering of these receptors is known to govern T-bet and CD11c expression in activated B cells.^51^ The elevated expression of T-bet in DN2 cells (and also ABCs) has been associated with development of autoimmunity and more specifically with autoreactive IgG production, enhanced antigen presentation to T cells and formation of spontaneous GCs.^13,38,39,52,53^ It is also important to mention that, in SLE mice models, conditional T-bet targeting in B cells leads to general improvement of health status,^54^ thus indicating that T-bet^+^ B cells are a pathogenetic population that may be targeted in clinical practice for therapeutic interventions.^34^ Moreover, considering the fact that the frequencies of these cells correlate with disease severity, we believe that they may also serve as prognostic and/or diagnostic markers of SLE.^34^

The fact that DN2 expansion is pronounced in African-American female SLE patients, indicates that genetic burden, as well as the sex of individuals, are crucial factors for the generation of these DN cells. Of note, TLR7 gene, which is of high importance for DN2 activation,^13^ is a chromosome X-linked gene, thus explaining (at least in part) the sex-bias that accompanies SLE immunopathogenesis.^55,56^ As far as genetic burden is concerned, it is well known that ethnicity -among other factors- is linked to the severity of SLE manifestations.^57^ A better understanding of the differences among the various ethnic groups could probably enable better management of SLE patients. In order to highlight this, we find it important to mention that in a clinical trial, in which IFNγ was targeted via an anti-IFNγ monoclonal antibody, no therapeutic impacts were produced in a cohort of non-African-American SLE patients with lupus nephritis.^58^ IFNγ is also of high importance for the activation of DN2 cells and, since these cells are more marked in African-American SLE patients, we consider the fact that no therapeutic impacts were observed in non-African-American patients as unsurprising and expected.^13,58^

### DN2 B cells in COVID-19

The function of DN B cells, both in health and disease, but mainly related to infectious diseases, is not yet fully elucidated and thorough investigation is required in order to understand the importance of DN percentage alterations that occur during pathological conditions. In case of COVID-19, our knowledge regarding DN B cells is extremely limited, as the disease was first observed almost two years ago.

Accumulating evidence, arising from a few studies that focused on B cell populations in COVID-19, suggest that DN2 B cells, along with the newly discovered DN3 subset, expand in severe and/or critical cases of the disease,^7,12,14,15^ while on the other hand, DN1 cells exhibit significant reductions.^7,8^ According to longitudinal progression studies though, the frequencies of these cells and the DN1/DN2 ratio are switching back to their initial levels (meaning the frequencies and ratio reported in healthy individuals), as the resolution of the disease approaches.^6^

In hospitalised COVID-19 patients, DN2 cells are associated with high neutralising antibody titres and elevated plasma concentrations of inflammatory biomarkers, such as CRP, CXCL10, and IL-6. ^7^ However, in general, it is not clear whether these cells are playing an active role in disease pathogenesis, or whether they just serve as bio-markers of the strong inflammation that occurs. The fact that T-bet expression in DN2 B cells from COVID-19 patients was found to be reduced, as compared to healthy individuals,^6^ indicates that the scenario of an active pathogenetic role for these cells in the aforementioned disease is not the most plausible. Nevertheless, some direct pathogenetic effects of DN2 B cells in COVID-19 could probably include extrafollicular B cell cytokine productions, enhanced antigen presentation to T cells and of course (auto)antibody production.^15^

Although we focus on DN2 cells here, as they are the DN subset that is also (and mainly) marked in rheumatic diseases,^13^ we find it inevitable not to make a few comments regarding DN3 cells. The fraction of DN3 cells seems to be by far the most altered in COVID-19, since their frequency was seen to significantly increase as disease severity increased.^8^ Moreover, in contrast to other DN subsets, DN3 showed significant negative correlation with ventilator parameters, such as SpO_2_ and PaO2/FiO_2_,^8^ thus indicating that in hypoxic environments, the development program tilts toward DN3 cells. Interestingly once again, in comparison to the other two DN subsets, DN3 showed more correlation with several laboratory parameters including leukocytes, neutrophils, markers of increased cellular turnover such as CPK and LDH, and others related to critical COVID-19.^8^ Of note, DN3 cells were related to predominant extrafollicular B cell responses, as they are considered as newly recruited extrafollicular B cells with low somatic mutations and low affinity maturation. ^7^

## MANAGEMENT OF RHEUMATIC DISEASES DURING THE COVID-19 PANDEMIC: POTENTIAL EFFECTS OF DN B CELLS

The symptomatology of COVID-19, as well as the potential outcome of death, have given rise to a worldwide concern and scare of exposition to SARS-CoV-2, especially among the rheumatological patients who believe to be at higher risk due to their immunological background and the immunosuppressive therapies. Nevertheless, contrary to these patients’ thoughts, it is strongly recommended by their caring physicians that all their therapies should be continued, as there is no convincing evidence so far that patients with rheumatic diseases are truly at higher risk than others.^59^

We strongly believe that the clarification of DN B cells’ role and function, especially for DN2 cells as they are an overlapping population in rheumatic diseases and COVID-19, can lead to better management of rheumatological patients during the pandemic. Of course, there are some major issues that -in our opinion- require investigation, in order to make good use of our knowledge regarding DN B cells and thus manage the rheumatic disease patients more wisely.

The first one of these major issues refers to the activation pathway of COVID-19 and more specifically to all the phases of the infection, from the moment SARS-CoV-2 invades the host to the moment cytokine release syndrome occurs, as a result of inefficacious viral response and host inflammatory response.^2,3,4^ The synthesis of pro-inflammatory cytokines promotes the recruitment of macrophages and neutrophils into the lung alveoli, thus inducing a hyper-inflammation.^60,61^ We mentioned before that we cannot say for sure whether DN2 cells play an active role in these processes or whether they are just a consequence of the inflammation. However, we find it interesting that TLR7, as well as IL-6, IFNα, IFNγ, TNFα, and other DN related cytokines or chemokines are involved in the pathway.^59^ Of note, TLR7 hyper-responsiveness is considered as crucial in the promotion of pathology mediated by IL-21,^13^ IL6 and IFNα.^62^ Also, the elevated soluble mediators in severe COVID-19, such as IL-6, CXCL10 and TNFα, may suppress GC reactions, and thus promote extrafollicular pathways.^63,64,65^

The second major issue that requires investigation refers to the impact of anti-rheumatic therapies, such as biologics, on the immunological memory achieved by COVID-19 vaccination or even resolved infection. Rituximab, an anti-CD20 chimeric monoclonal antibody used in battle against RA,^66^ as well as in management of lymphoma,^67^ significantly increases the risk of HBV-reactivation in resolved patients and of viraemic flares in chronically infected patients with low viral loads.^68,69^ Of course, even so, long-lived ASCs which lack CD20 expression, are thought to be preserved and thus maintain serum antibody responses in the absence of memory B cells.^70^ However, we cannot guarantee that under these circumstances ASCs can be reliably formed following HBV infection. In a similar way to HBV infection, we wonder whether, and to what extent, B cell depletion via pharmacological agents, such as Rituximab, Belimumab and others,^66,71^ affects the immunological memory achieved by COVID-19 vaccination or infection. In addition to this, there is also something else that is of high importance. DN2 cells expand in severe and/or critical cases of COVID-19, in contrast to more mild cases.^7,12,14,15^ However, we do not know whether a COVID-19 vaccination, which we do not consider as a severe or critical COVID-19 case, induces DN2 expansion. Of note, an increase in circulating DN B cells has been observed after vaccination against influenza virus in healthy subjects.^72^ Finding the answers to these questions, probably could enable better management of rheumatological patients during the pandemic, as determining the most proper vaccination and medication time points for the patients, would bring the most effective therapeutic benefits.

The third major issue, which is the last we want to discuss about, but not the least important, refers to pharmacological agents, approved for anti-rheumatic therapies and now also registered in clinical trials to treat COVID-19. Interestingly, some of this medication, such as tocilizumab, an IL-6 receptor antagonist, is known to affect DN B cell percentages in RA,^73^ while others such as baricitinib, a JAK1 and JAK2 inhibitor,^74^ can target the IL-12-STAT4 axis which is the most potent at inducing both IFNγ and IL-21 by human CD4+ T cells, the cytokines promoting the differentiation of DN B cells in human SLE.^75^ Taking into account this evidence, we believe that targeting DN B cells (especially DN2 and DN3) may contribute to the management of rheumatic diseases during COVID-19 era, and even become a successful therapeutic approach for the infection.

## ABBREVIATIONS

ABCs: Age-associated B cells
ARDS: Acute respiratory distress syndrome
ASC: Antibody-secreting cell
COVID-19: Coronavirus disease 2019
CVID: Common variable immunodeficiency
CytoFOX: Cytoplasmic FOXO1
DN: Double negative B cells
DN2: Double negative 2 B cell subset
GC: Germinal center
MS: Multiple sclerosis
NSCLC: Non-small cell lung cancer
pSS: primary Sjögren’s syndrome
RA: Rheumatoid arthritis
SARS-CoV-2: Severe acute respiratory syndrome coronavirus 2
SCD: Scleroderma
SLE: Systemic lupus erythematosus

## References

[1] Zhu N Zhang D Wang W Li X Yang B Song J A novel Coronavirus from patients with pneumonia in China, 2019. N Engl J Med 2020;382(8):727–33. 31978945 10.1056/NEJMoa2001017 PMC7092803

[2] Vabret N Britton GJ Gruber C Hegde S Kim J Kuksin M Immunology of COVID-19: Current state of the science. Immunity 2020;52(6):910–41. 32505227 10.1016/j.immuni.2020.05.002 PMC7200337

[3] Zhou Y Fu B Zheng X Wang D Zhao C Qi Y Pathogenic T-cells and inflammatory monocytes incite inflammatory storms in severe COVID-19 patients. Natl Sci Rev 2020;7(6):998–1002. 34676125 10.1093/nsr/nwaa041 PMC7108005

[4] Gustine JN Jones D. Immunopathology of hyperinflammation in COVID-19. Am J Pathol 2021;191(1):4–17. 32919977 10.1016/j.ajpath.2020.08.009 PMC7484812

[5] Wu C Chen X Cai Y Xia J Zhou X Xu S Risk factors associated with acute respiratory distress syndrome and death in patients with Coronavirus disease 2019 pneumonia in Wuhan, China. JAMA Intern Med 2020;180(7):934–43. 32167524 10.1001/jamainternmed.2020.0994 PMC7070509

[6] Wildner NH Ahmadi P Schulte S Brauneck F Kohsar M Lütgehetmann M B cell analysis in SARS-CoV-2 versus malaria: Increased frequencies of plasmablasts and atypical memory B cells in COVID-19. J Leukoc Biol 2021;109(1):77–90. 33617048 10.1002/JLB.5COVA0620-370RR PMC10016889

[7] Woodruff MC Ramonell RP Nguyen DC Cashman KS Saini AS Haddad NS Extrafollicular B cell responses correlate with neutralizing antibodies and morbidity in COVID-19. Nat Immunol 2020;21(12):1506–16. 33028979 10.1038/s41590-020-00814-z PMC7739702

[8] Sosa-Hernández VA Torres-Ruíz J Cervantes-Díaz R Romero-Ramírez S Páez-Franco JC Meza-Sánchez DE B cell subsets as severity-associated signatures in COVID-19 patients. Front Immunol 2020;11:611004. 33343585 10.3389/fimmu.2020.611004 PMC7744304

[9] Burton AR Maini MK. Human antiviral B cell responses: Emerging lessons from hepatitis B and COVID-19. Immunol Rev 2021;299(1):108–17. 33559128 10.1111/imr.12953 PMC8014162

[10] Moir S Fauci AS. B-cell responses to HIV infection. Immunol Rev 2017;275(1):33–48. 28133792 10.1111/imr.12502 PMC5300048

[11] Silveira ELV Dominguez MR Soares IS. To B or not to B: Understanding B cell responses in the development of malaria infection. Front Immunol 2018;9:2961. 30619319 10.3389/fimmu.2018.02961 PMC6302011

[12] Oliviero B Varchetta S Mele D Mantovani S Cerino A Perotti CG Expansion of atypical memory B cells is a prominent feature of COVID-19. Cell Mol Immunol 2020;17(10):1101–3. 32879471 10.1038/s41423-020-00542-2 PMC7463104

[13] Jenks SA Cashman KS Zumaquero E Marigorta UM Patel AV Wang X Distinct Effector B Cells Induced by Unregulated Toll-like Receptor 7 Contribute to Pathogenic Responses in Systemic Lupus Erythematosus. Immunity 2018;49(4):725–739.e6. 30314758 10.1016/j.immuni.2018.08.015 PMC6217820

[14] Woodruff M Ramonell R Cashman K Nguyen D Ley A Kyu S Critically ill SARS-CoV-2 patients display lupus-like hallmarks of extrafollicular B cell activation. medRxiv [Internet]. 2020; Available from: 10.1101/2020.04.29.20083717

[15] Farris AD Guthridge JM. Overlapping B cell pathways in severe COVID-19 and lupus. Nat Immunol 2020;21(12):1478–80. 33139917 10.1038/s41590-020-00822-z

[16] Colonna-Romano G Bulati M Aquino A Pellicano M Vitello S Lio D A double-negative (IgD-CD27-) B cell population is increased in the peripheral blood of elderly people. Mech Ageing Dev 2009;130(10):681–90. 19698733 10.1016/j.mad.2009.08.003

[17] Wei C Anolik J Cappione A Zheng B Pugh-Bernard A Brooks J A new population of cells lacking expression of CD27 represents a notable component of the B cell memory compartment in systemic lupus erythematosus. J Immunol 2007;178(10):6624–33. 17475894 10.4049/jimmunol.178.10.6624

[18] Claes N Fraussen J Vanheusden M Hellings N Stinissen P Van Wijmeersch B Age-associated B cells with proinflammatory characteristics are expanded in a proportion of multiple sclerosis patients. J Immunol 2016;197(12):4576–83. 27837111 10.4049/jimmunol.1502448

[19] Moir S Ho J Malaspina A Wang W DiPoto AC O’Shea MA Evidence for HIV-associated B cell exhaustion in a dysfunctional memory B cell compartment in HIV-infected viremic individuals. J Exp Med 2008;205(8):1797–805. 18625747 10.1084/jem.20072683 PMC2525604

[20] Portugal S Tipton CM Sohn H Kone Y Wang J Li S Malaria-associated atypical memory B cells exhibit markedly reduced B cell receptor signaling and effector function. Elife [Internet]. 2015;4. Available from: 10.7554/eLife.07218 PMC4444601 25955968

[21] Wang S Wang J Kumar V Karnell JL Naiman B Gross PS IL-21 drives expansion and plasma cell differentiation of autoreactive CD11chiT-bet+ B cells in SLE. Nat Commun 2018; 9(1):1758. 29717110 10.1038/s41467-018-03750-7 PMC5931508

[22] Zumaquero E Stone SL Scharer CD Jenks SA Nellore A Mousseau B IFNγ induces epigenetic programming of human T-bethi B cells and promotes TLR7/8 and IL-21 induced differentiation. E life 2019;8:e41641. 31090539 10.7554/eLife.41641 PMC6544433

[23] Tipton CM Fucile CF Darce J Chida A Ichikawa T Gregoretti I Diversity, cellular origin and autoreactivity of antibody-secreting cell population expansions in acute systemic lupus erythematosus. Nat Immunol 2015;16(7):755–65. 26006014 10.1038/ni.3175 PMC4512288

[24] Frasca D Diaz A Romero M Blomberg BB. Phenotypic and functional characterization of Double Negative B cells in the blood of individuals with obesity. Front Immunol 2021;12:616650. 33708209 10.3389/fimmu.2021.616650 PMC7940530

[25] Frasca D Diaz A Romero M Thaller S Blomberg BB. Metabolic requirements of human pro-inflammatory B cells in aging and obesity. PLoS One 2019;14(7):e0219545. 31287846 10.1371/journal.pone.0219545 PMC6615614

[26] Centuori SM Gomes CJ Kim SS Putnam CW Larsen BT Garland LL Double-negative (CD27−IgD−) B cells are expanded in NSCLC and inversely correlate with affinity-matured B cell populations. J Transl Med[Internet] 2018;16(1). Available from: 10.1186/s12967-018-1404-z PMC5815250 29448960

[27] Maul RW Catalina MD Kumar V Bachali P Grammer AC Wang S Transcriptome and IgH repertoire analyses show that CD11chi B cells are a distinct population with similarity to B cells arising in autoimmunity and infection. Front Immunol [Internet] 2021;12. Available from: 10.3389/fimmu.2021.649458 PMC8017342 33815408

[28] Rubtsov AV Marrack P Rubtsova K. T-bet Expressing B cells – novel target for autoimmune therapies? Cell Immunol 2017;321:35–9. 28641866 10.1016/j.cellimm.2017.04.011

[29] Barnett BE Staupe RP Odorizzi PM Palko O Tomov VT Mahan AE Cutting Edge: B Cell-Intrinsic T-bet Expression Is Required To Control Chronic Viral Infection. J Immunol 2016;197(4):1017–22. 27430722 10.4049/jimmunol.1500368 PMC4975981

[30] Richardson CT Slack MA Dhillon G Marcus CZ Barnard J Palanichamy A Failure of B Cell Tolerance in COVID. Front Immunol 2019;10:2881 31921145 10.3389/fimmu.2019.02881 PMC6914825

[31] Hritzo Ahye MK Golding A. Cytoplasmic FOXO1 identifies a novel disease-activity associated B cell phenotype in SLE. Lupus Sci Med 2018;5(1):e000296. 30397498 10.1136/lupus-2018-000296 PMC6203050

[32] Sander S Chu VT Yasuda T Franklin A Graf R Cakado DP PI3 Kinase and FOXO1 Transcription Factor Activity Differentially Control B Cells in the Germinal Center Light and Dark Zones. Immunity 2015;43(6):1075–86. 26620760 10.1016/j.immuni.2015.10.021

[33] Luo W Weisel F Shlomchik MJ. B Cell Receptor and CD40 Signaling Are Rewired for Synergistic Induction of the c-Myc Transcription Factor in Germinal Center B Cells. Immunity 2018;48(2):313–326.e5. 29396161 10.1016/j.immuni.2018.01.008 PMC5821563

[34] Sachinidis A Xanthopoulos K Garyfallos A. Age-associated B cells (ABCs) in the prognosis, diagnosis and therapy of Systemic Lupus Erythematosus (SLE). Mediterr J Rheumatol 2020;31(3):311–8. 33163863 10.31138/mjr.31.3.311 PMC7641025

[35] Rinaldi S Pallikkuth S George VK de Armas LR Pahwa R Sanchez CM Paradoxical aging in HIV: immune senescence of B Cells is most prominent in young age. Aging (Albany NY) 2017;9(4):1307–25. 28448963 10.18632/aging.101229 PMC5425129

[36] Illingworth J Butler NS Roetynck S Mwacharo J Pierce SK Bejon P Chronic exposure to Plasmodium falciparum is associated with phenotypic evidence of B and T cell exhaustion. J Immunol 2013;190(3):1038–47. 23264654 10.4049/jimmunol.1202438 PMC3549224

[37] Hao Y O’Neill P Naradikian MS Scholz JL Cancro MP. A B-cell subset uniquely responsive to innate stimuli accumulates in aged mice. Blood 2011;118(5):1294–304. 21562046 10.1182/blood-2011-01-330530 PMC3152496

[38] Rubtsov AV Rubtsova K Fischer A Meehan RT Gillis JZ Kappler JW Toll-like receptor 7 (TLR7)-driven accumulation of a novel CD11c⁺ B-cell population is important for the development of autoimmunity. Blood 2011;118(5):1305–15. 21543762 10.1182/blood-2011-01-331462 PMC3152497

[39] Rubtsov AV Marrack P Rubtsova K. T-bet Expressing B cells – novel target for autoimmune therapies? Cell Immunol 2017;321:35–9. 28641866 10.1016/j.cellimm.2017.04.011

[40] Rubtsova K Rubtsov AV van Dyk LF Kappler JW Marrack P. T-box transcription factor T-bet, a key player in a unique type of B-cell activation essential for effective viral clearance. Proc Natl Acad Sci U S A. 2013;110(34):E3216–E3224 23922396 10.1073/pnas.1312348110 PMC3752276

[41] Rubtsova K Rubtsov AV Halemano K Li SX Kappler JW Santiago ML T Cell Production of IFNγ in Response to TLR7/IL-12 Stimulates Optimal B Cell Responses to Viruses. PLoS One 2016;11(11):e0166322 27880772 10.1371/journal.pone.0166322 PMC5120817

[42] Phalke S Marrack P. Age (autoimmunity) associated B cells (ABCs) and their relatives. Curr Opin Immunol 2018;55:24–30. 10.1016/j.coi.2018.09.007 30388513

[43] Cancro MP. Age-associated B cells. Annu Rev Immunol 2020;38(1):315–40. 31986068 10.1146/annurev-immunol-092419-031130

[44] Mesin L Ersching J Victora GD. Germinal center B cell dynamics. Immunity 2016;45(3):471–82. 27653600 10.1016/j.immuni.2016.09.001 PMC5123673

[45] Allman D Wilmore JR Gaudette BT. The continuing story of T-cell independent antibodies. Immunol Rev 2019;288(1):128–35. 30874357 10.1111/imr.12754 PMC6653682

[46] Lam JH Smith FL Baumgarth N. B cell activation and response regulation during viral infections. Viral Immunol 2020;33(4):294–306. 32326852 10.1089/vim.2019.0207 PMC7247032

[47] Elsner RA Shlomchik MJ. Germinal center and extrafollicular B cell responses in vaccination, immunity, and autoimmunity. Immunity 2020;53(6):1136–50. 33326765 10.1016/j.immuni.2020.11.006 PMC7748291

[48] Jenks SA Cashman KS Woodruff MC Lee FE-H Sanz I. Extrafollicular responses in humans and SLE. Immunol Rev 2019;288(1):136–48. 30874345 10.1111/imr.12741 PMC6422038

[49] Rao DA Gurish MF Marshall JL Slowikowski K Fonseka CY Liu Y Pathologically expanded peripheral T helper cell subset drives B cells in rheumatoid arthritis. Nature 2017;542(7639):110–4. 28150777 10.1038/nature20810 PMC5349321

[50] Cunningham AF Gaspal F Serre K Mohr E Henderson IR Scott-Tucker A Salmonella induces a switched antibody response without germinal centers that impedes the extracellular spread of infection. J Immunol 2007;178(10):6200–7. 17475847 10.4049/jimmunol.178.10.6200

[51] Naradikian MS Myles A Beiting DP Roberts KJ Dawson L Herati RS Cutting edge: IL-4, IL-21, and IFN-γ interact to govern T-bet and CD11c expression in TLR-activated B cells. J Immunol 2016;197(4):1023–8. 27430719 10.4049/jimmunol.1600522 PMC4975960

[52] Rubtsov AV Rubtsova K Kappler JW Jacobelli J Friedman RS Marrack P. CD11c-Expressing B Cells Are Located at the T Cell/B Cell Border in Spleen and Are Potent APCs. J Immunol 2015;195(1):71–9. 26034175 10.4049/jimmunol.1500055 PMC4475418

[53] Domeier PP Chodisetti SB Soni C Schell SL Elias MJ Wong EB IFN-γ receptor and STAT1 signaling in B cells are central to spontaneous germinal center formation and autoimmunity. J Exp Med 2016;213(5):715–32. 27069112 10.1084/jem.20151722 PMC4854731

[54] Rubtsova K Rubtsov AV Thurman JM Mennona JM Kappler JW Marrack P. B cells expressing the transcription factor T-bet drive lupus-like autoimmunity. J Clin Invest 2017;127(4):1392–404. 28240602 10.1172/JCI91250 PMC5373868

[55] Ricker E Manni M Flores-Castro D Jenkins D Gupta S Rivera-Correa J Sex-specific differences in the function and differentiation of ABCs mark TLR7-driven immunopathogenesis [Internet]. bioRxiv 2021. Available from: 10.1101/2021.01.20.427400

[56] Rubtsova K Marrack P Rubtsov AV. TLR7, IFNγ, and T-bet: their roles in the development of ABCs in female-biased autoimmunity. Cell Immunol 2015;294(2):80–3. 25541140 10.1016/j.cellimm.2014.12.002 PMC4380581

[57] González LA Toloza SM McGwin G Jr Alarcón GS. Ethnicity in systemic lupus erythematosus (SLE): its influence on susceptibility and outcomes. Lupus 2013;22(12):1214–24. 24097993 10.1177/0961203313502571

[58] Boedigheimer MJ Martin DA Amoura Z Sánchez-Guerrero J Romero-Diaz J Kivitz A Safety, pharmacokinetics and pharmacodynamics of AMG 811, an anti-interferon-γ monoclonal antibody, in SLE subjects without or with lupus nephritis. Lupus Sci Med 2017;4(1):e000226. 29018537 10.1136/lupus-2017-000226 PMC5604705

[59] Gremese E Ferraccioli ES Alivernini S Tolusso B Ferraccioli G. Basic immunology may lead to translational therapeutic rationale: SARS-CoV-2 and rheumatic diseases. Eur J Clin Invest 2020;50(9):e13342. 32645207 10.1111/eci.13342 PMC7404583

[60] Ritter M Mennerich D Weith A Seither P. Characterization of Toll-like receptors in primary lung epithelial cells: strong impact of the TLR3 ligand poly(I:C) on the regulation of Toll-like receptors, adaptor proteins and inflammatory response. J Inflamm (Lond) 2005;2(1):16. 16316467 10.1186/1476-9255-2-16 PMC1315317

[61] Channappanavar R Perlman S. Pathogenic human coronavirus infections: causes and consequences of cytokine storm and immunopathology. Semin Immunopathol 2017;39(5):529–39. 28466096 10.1007/s00281-017-0629-x PMC7079893

[62] Jego G Palucka AK Blanck J-P Chalouni C Pascual V Banchereau J. Plasmacytoid dendritic cells induce plasma cell differentiation through type I interferon and interleukin 6. Immunity 2003;19(2):225–34. 12932356 10.1016/s1074-7613(03)00208-5

[63] Kopf M Herren S Wiles MV Pepys MB Kosco-Vilbois MH. Interleukin 6 influences germinal center development and antibody production via a contribution of C3 complement component. J Exp Med 1998;188(10):1895–906. 9815267 10.1084/jem.188.10.1895 PMC2212418

[64] Ly A Liao Y Pietrzak H Ioannidis LJ Sidwell T Gloury R Transcription factor T-bet in B cells modulates germinal center polarization and antibody affinity maturation in response to malaria. Cell Rep 2019;29(8):2257–2269.e6. 31747599 10.1016/j.celrep.2019.10.087

[65] Kaneko N Kuo H-H Boucau J Farmer JR Allard-Chamard H Mahajan VS Loss of Bcl-6-expressing T follicular helper cells and germinal centers in COVID-19. Cell 2020;183(1):143–157.e13. 32877699 10.1016/j.cell.2020.08.025 PMC7437499

[66] Cohen MD Keystone E. Rituximab for rheumatoid arthritis. Rheumatol Ther 2015;2(2):99–111. 27747531 10.1007/s40744-015-0016-9 PMC4883263

[67] Dotan E Aggarwal C Smith MR. Impact of rituximab (Rituxan) on the treatment of B-cell non-Hodgkin’s lymphoma. P T 2010;35(3):148–57. 20442809 PMC2844047

[68] Loomba R Liang TJ. Hepatitis B reactivation associated with immune suppressive and biological modifier therapies: Current concepts, management strategies, and future directions. Gastroenterology 2017;152(6):1297–309. 28219691 10.1053/j.gastro.2017.02.009 PMC5501983

[69] Shouval D Shibolet O. Immunosuppression and HBV reactivation. Semin Liver Dis 2013;33(2):167–77. 23749673 10.1055/s-0033-1345722

[70] Hammarlund E Thomas A Amanna IJ Holden LA Slayden OD Park B Plasma cell survival in the absence of B cell memory. Nat Commun 2017;8(1):1781. 29176567 10.1038/s41467-017-01901-w PMC5701209

[71] Ramsköld D Parodis I Lakshmikanth T Sippl N Khademi M Chen Y B cell alterations during BAFF inhibition with belimumab in SLE. EBioMedicine 2019;40:517–527 30593436 10.1016/j.ebiom.2018.12.035 PMC6412067

[72] Ruschil C Gabernet G Lepennetier G Heumos S Kaminski M Hracsko Z Specific induction of double negative B cells during protective and pathogenic immune responses. Front Immunol 2020;11:606338. 33391273 10.3389/fimmu.2020.606338 PMC7775384

[73] Moura RA Quaresma C Vieira AR Gonçalves MJ Polido-Pereira J Romão VC B-cell phenotype and IgD-CD27- memory B cells are affected by TNF-inhibitors and tocilizumab treatment in rheumatoid arthritis. PLoS One 2017;12(9):e0182927. 28886017 10.1371/journal.pone.0182927 PMC5590747

[74] Al-Salama ZT Scott LJ. Baricitinib: A review in rheumatoid arthritis. Drugs 2018;78(7):761–72. 29687421 10.1007/s40265-018-0908-4

[75] Ueno H. The IL-12-STAT4 axis in the pathogenesis of human systemic lupus erythematosus. Eur J Immunol 2020;50(1):10–6. 31762023 10.1002/eji.201948134

